# Epidemiology and management of urological emergencies in a tertiary care setting in Scandinavia

**DOI:** 10.1186/s12245-025-00882-8

**Published:** 2025-04-15

**Authors:** Adin Osmancevic, Alma Petersson, Anna Duverin, Bina Merzaai, Ella Hedlund, Giovanni Morera Porras, Isabella Albinsson, Jasmine AL-Hadad, Salome Olsson, Daniel Vestberg, Erik Sagen, Suleiman Abuhasanein

**Affiliations:** 1 Department of Surgery, Urology section, NU Hospital Group, Region Västra Götaland, Uddevalla, Sweden; 2 Närhälsan Torslanda Health centre, Gothenburg, Sweden; 3 Department of gynaecology/obstetrics, Sahlgrenska University Hospital, Region Västra Götaland, Gothenburg, Sweden; 4 Närhälsan Högsbo health center, Gothenburg, Sweden; 5 Department of Emergency Medicine, NU Hospital Group, Region Västra Götaland, Trollhättan, Sweden; 6 Department of gynecology/obstetrics, NU-Hospital Group, Trollhättan, Sweden; 7 Department of pediatrics, Queen Silvia Children’s Hospital, Sahlgrenska University Hospital, Gothenburg, Sweden; 8 Department of Research and Development, NU-Hospital Group, Trollhättan, Sweden; 9 Department of Urology, Institute of Clinical Science, Sahlgrenska Academy, University of Gothenburg, Gothenburg, 413 90 Sweden

**Keywords:** Catheter, Computed tomography, Emergency unit, Hematuria, Loin pain, Primary care healthcare, Ultrasound, Urinary tract infection, Urological emergencies

## Abstract

**Objective:**

To develop a baseline database detailing the distribution of urological emergencies and to define their epidemiological profile in a tertiary care setting, with the hope of providing important data for health planning.

**Design, settings and participants:**

A retrospective study was conducted on all patients presenting with urological emergencies at the Emergency Department (ED) of the NU Hospital Group in Trollhättan, Sweden throughout 2019. Medical records of identified patients were reviewed retrospectively to summarize pertinent information.

**Main results:**

In 2019, 2 433 patients visited the ED with urological complaints, with 71% being male. Most patients (83%) were self-referred and 15% referred by general practitioners (GPs). Loin pain, infectious symptoms, and lower urinary tract symptoms were the most common complaints. Urinary and genital infections (UGIs) were the most frequent diagnoses (37%), followed by urolithiasis (24%). 28% of patients required admission, particularly for UGIs (42%). Self-referred patients had a higher admission rate compared to those referred by GPs. Radiological investigations were performed in 48% of cases, though 65% showed no urological pathology.

**Conclusions:**

Most patients self-referred to the ED, and many required hospitalization, particularly for UGIs. Enhancing the management of urological emergencies in primary care and refining guidelines for acute imaging could contribute to more efficient use of healthcare resources.

## Introduction

Urological emergencies, while diverse in presentation, impose a significant medical and socioeconomic burden on healthcare providers [1]. These emergencies encompass a broad spectrum of conditions, ranging from acute urinary retention (AUR) to urological malignancies which can result in substantial morbidity and mortality. Urological emergencies remain a substantial aspect of daily clinical practice [2]. They account for up to 27% of all urological admissions in tertiary care institutions [3].

These emergencies affect patients across all age groups and genders, presenting with a wide range of conditions, including benign, malignant, acute, chronic, traumatic, postoperative, reconstructive, rare, and common pathologies [4, 5]. Additionally, certain urological emergencies exhibit seasonal variations, and the type of medical staff managing them in emergency departments (EDs) varies across regions [6, 7].

A trend of increasing emergency visits has been noticed [8]. The knowledge of the distribution of acute urological conditions is vital for a successful care service delivery. However, there is a relative paucity of information about the panorama of the urological emergencies [9]. A better understanding of the commonly encountered acute urologic conditions is urgently needed.

To our knowledge, a large-scale epidemiological, population-based study embracing almost all urological emergencies has previously not been performed in Scandinavia. The purpose of this study is therefore to establish a baseline database on the epidemiological profile of the urological emergencies in a tertiary care hospital. This with the hope of providing important data for health care planning and subsequently improving patients care delivery through better decision making.

## Materials and methods

### Patients

This is a retrospective study of all new patients with urological emergencies presenting to our ED in the NU Hospital Group, Trollhättan, Sweden, between 1st January 2019 and 31st December 2019. This year was chosen with regards to the Covid-19 pandemic, which obviously affected clinical practice of the majority of urological units worldwide [10]. Research was carried out from our institution database, looking for the relevant urological diagnoses according to the International Classification of Diseases (ICD10) [11]. Diagnoses were established either in the ED if the patient was discharged or upon hospital admission and documented in the discharge summary.

The medical records of all selected patients were retrospectively reviewed to gather relevant information. This included patient characteristics, clinical and radiological findings, and details of their hospital stay. Outcomes were evaluated both within the first 24 h and at the one-month follow-up. The number of patient contacts with a urological unit, including both phone calls and in-person visits, within one year following the initial ED visit was recorded.

### Definitions

**Fig. 1:**
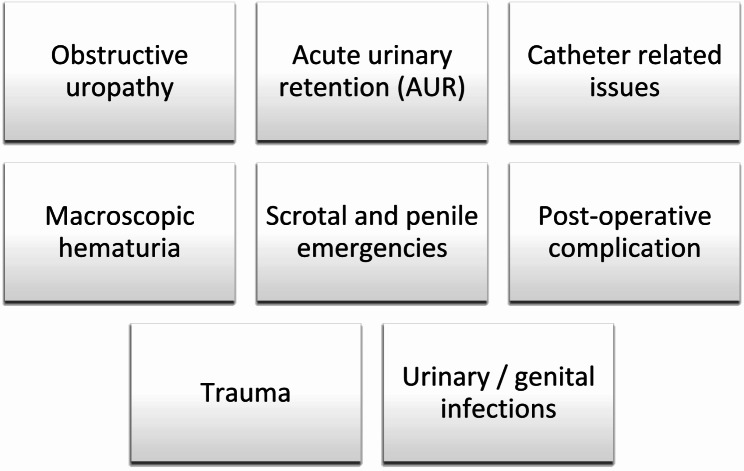
The main urological emergencies categories of patients presenting to the Emergency Department with urological complaints at the NU Hospital Group in Sweden in 2019

Urological emergencies in this study were categorized into eight main groups (Fig. 1). **Obstructive uropathy** involves severe and fluctuating pain caused by an upper urinary tract obstruction (e.g., renal colic), typically diagnosed using non-contrast enhanced computed tomography (CT) or abdominal ultrasound (US), or acute renal failure due to hydronephrosis, often resulting from locally advanced prostate cancer or an undiagnosed/untreated benign prostatic enlargement. Other asymptomatic obstructive uropathies are also included. **Acute urinary retention (AUR)** is characterized by a sudden, painful inability to urinate caused by functional or mechanical obstruction at the bladder outlet [12].

**Catheter-related issues** encompass complications such as pain, urgency, blockage, obstruction, dislodgement or leakage associated with urinary drainage devices, including pyelostomy, suprapubic catheters, double pigtail catheters, and indwelling catheters [13]. **Macroscopic hematuria** refers to visible blood in the urine. **Scrotal and penile emergencies** include non-traumatic conditions in this region, such as testicular torsion, Fournier’s gangrene, priapism, paraphimosis, phimosis, penile fracture and related pain (testalgia and scrotalgia) [14]. **Post-operative complications** involve patients presenting to the ED within 30 days of undergoing urological procedures with any complication such as infection, bleeding, pain etc. **Trauma** pertains to injuries affecting the urinary and genital systems in both males and females. **Urinary and genital infections (UGIs)** include infections of the urinary system in both genders, infections of the male genital system [15].

### Emergency department in the NU hospital group, Trollhättan, Sweden

The Emergency Department (ED) is responsible for providing medical and surgical care to patients requiring immediate attention. In 2019, the ED in NU Hospital Group (catchment 290 000 inhabitants) was divided into six sections: internal medicine, pediatrics, gynecology, orthopedics, ear, nose, and throat (ENT), and a section dedicated to surgical, urological, and trauma patients (SUT). The SUT section consists of a 13-bed unit and there are also four additional resus rooms available. The ED is open all hours of the day. Surgeons, emergency physicians, urologists and trainees assigned to the ED manage all urgent referrals and are equipped to perform minor procedures under local anesthesia. A senior urologist consultant is available on-call at all times to provide continuous support.

### Statistical analyses

Descriptive statistics were employed to summarize patient characteristics. The most common conditions were analyzed based on age and sex. Continuous variables were expressed as median and interquartile range (IQR). The diagnostic agreement variability between the initial preliminary diagnoses made in the ED and the final diagnoses established after radiology and laboratory tests was evaluated using Cohen’s kappa. All statistical analyses were conducted using SPSS software, version 29 (IBM Corp., Armonk, NY, USA).

## Results

**Fig. 2:**
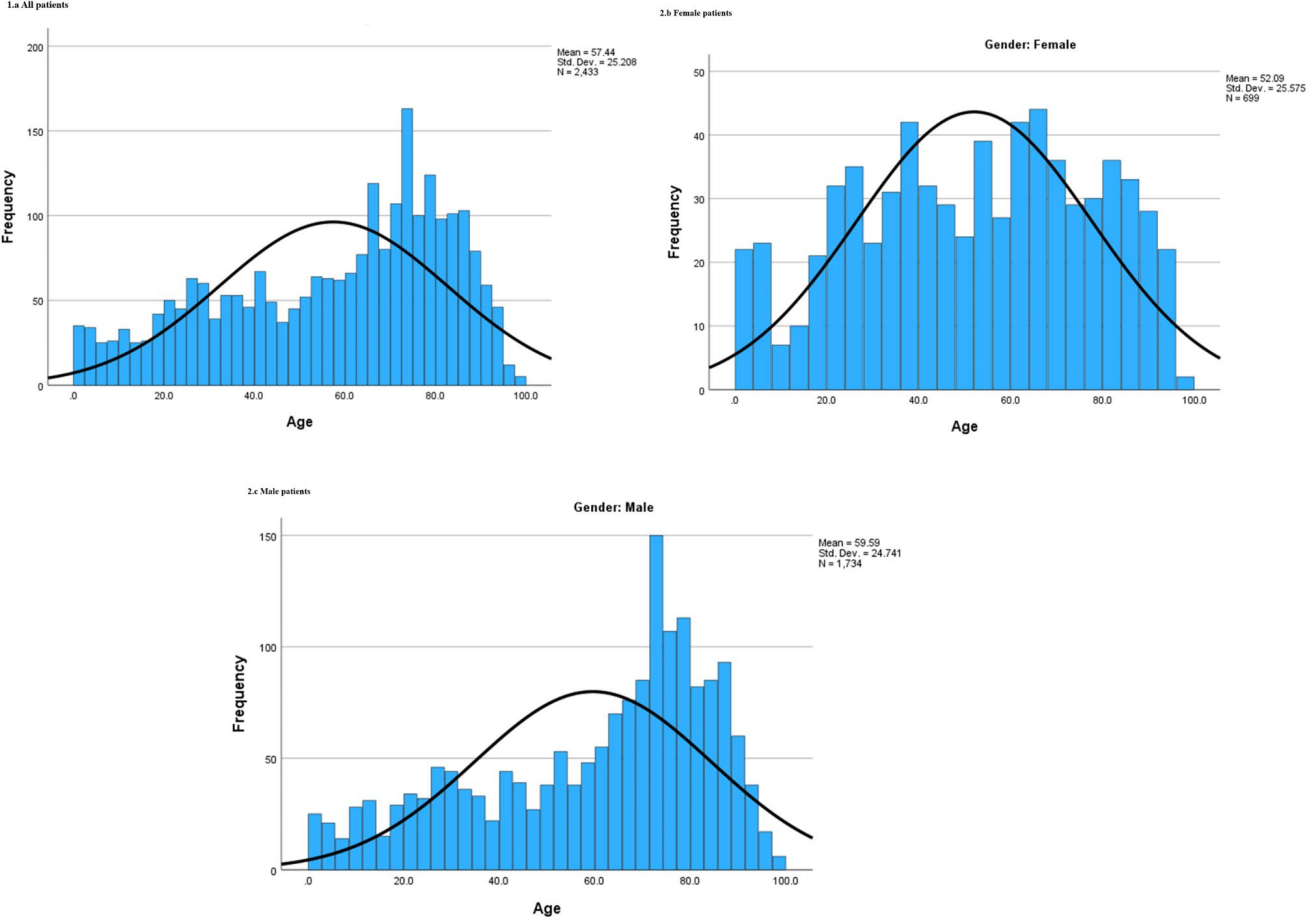
The age distribution of patients presenting to the Emergency Department with urological complaints at the NU Hospital Group in Sweden in 2019

In 2019, a total of 70 765 patients visited the ED, of which 2 433 (3.5%) presented with one or more urological complaints. Among them, 1 734 (71%) were male. The overall median age was 64 years (IQR 37–78). The age distribution varied between genders, with males having a higher median age of 67 years (IQR 42–79) than females 55 years (IQR 32–73). The age distribution of males was skewed towards older age groups, while the female age distribution was more evenly spread across middle and older ages (Fig. 2).

Of all patients presenting with urological complaints, 2 072 (85%) were triaged to the SUT section of the ED. A total of 2 018 patients (83%) self-referred to the ED (patients arrived at the ED either independently or via ambulance, without undergoing prior assessment by a general practitioner (GP)). Only 373 (15%) were referred by GPs. A total of 262 patients (11% of the entire cohort) presented with post-operative complications, which included infection, bleeding, wound infections, hematoma formation and urinary retention. These complications varied in severity and required appropriate management to ensure optimal recovery for the affected patients. Within one year of their initial ED visit, 755 patients (31%) had one or more follow-up contacts with the urology unit related to their initial ED visit. (Table 1)

**Table 1:** Demographic and clinical characteristics of patients presenting with urological complaints to the Emergency Department at the NU Hospital Group in Sweden in 2019. Figures represent the number of patients (% of the row) if not otherwise indicated. (CT: computed tomography, IQR: interquartile range, SUT: surgical, urological, and trauma)

Variable name		All
Gender	Male	1734 (71)
Age	Years (IQR)	64 (37–78)
Triage section	SUT	2072 (85)
	Internal medicine	260 (11)
	Other sections	101 (4)
Mode of presentation	Self-referral	2018 (83)
	Referred by primary care	373 (15)
	Others*	42 (2)
Post-op complication	Yes	262 (11)
Catheterised patient**	Yes	491 (20)
Catheter type**	Indwelling catheter	251 (10)
	Pyelostomy	127 (5)
	Suprapubic catheter	82 (3)
	Double pigtail catheter	40 (2)
Admission	All patients	675 (28)
	Self-referral	583 (29)
	Referred by primary care	78 (21)
Admission length	Days (IQR)	4 (3–6)
Admission department	Urology department	281 (65)
Patients undergone acute procedure		215 (9)
Acute radiology	All	712 (29)
	Abdominal CT	464 (65)
	Abdominal ultrasound	156 (22)
Acute treatment	With antibiotics (AB)	999 (41)
AB treatment length	Days (IQR)	8 (5–10)
Blood transfusion		24 (1)
Number of contacts***	1–4	649 (27)
	> 4	106 (4)

* For example, a referral from other hospitals

** Patients presenting to the emergency department who already have any type of urological catheter in place

*** Number of contacts with urological unit within one year after the initial contact with ED

### Symptoms, diagnoses, and admission rates

A total of 515 patients (21%) presented with loin pain, 421 patients (17%) had UGI symptoms such as fever, chills and rigors, fatigue, malaise, or pain, swelling and redness of scrotum. 255 patients (11%) presented with lower urinary tract symptoms (LUTS), and 247 (10%) experienced AUR. A total of 262 patients (11%) presented with macroscopic hematuria. All cases with macroscopic hematuria were referred to a urology unit for further evaluation, following national guidelines, which included cystoscopy and computed tomography urography [16]. No seasonal variation in these urological complaints was observed.

UGIs were the most common diagnoses, accounting for 904 patients (37%), followed by urolithiasis as the second most frequent diagnosis, with 586 patients (24%). The variability in diagnostic agreement between the initial diagnosis made by ED staff and the final diagnosis, as measured by Cohen’s kappa, was κ = 0.741 (*p* < 0.001). The various diagnoses for patients presenting with urological symptoms to the ED at the NU Hospital Group in Sweden in 2019 are detailed in Table 2.

**Table 2:** The various diagnoses and admission rates for patients presenting with urological complaints to the Emergency Department at the NU Hospital Group in Sweden in 2019. Figures represent number of patients (% of the row) if not otherwise indicated. (AUR: acute urinary retention, LUTS: lower urinary tract symptoms)

Diagnosis	Admission	Discharge	Total	*P* value
Urinary and genital infections	285 (32)	619 (68)	904	< 0.001*
Urolithiasis	114 (20)	472 (80)	586	< 0.001
AUR	44 (13)	288 (87)	332	< 0.001
Macroscopic hematuria	126 (48)	139 (52)	265	< 0.001
Scrotal and penile issues	14 (12)	99 (88)	113	< 0.001
Uro cancer	46 (84)	9 (16)	55	< 0.001
Catheter related issues	17 (33)	34 (67)	51	0.368
LUTS	3 (8)	35 (92)	38	0.006
Trauma	4 (18)	18 (82)	22	0.314
Others**	22 (33)	45 (67)	67	0.345
Total	675 (28)	1758 (72)	2433	

* Comparison of the category in the row as a group with all other categories in the cohort as another group

** Included non-specific abdominal pain, postoperative complications

**Fig. 3:**
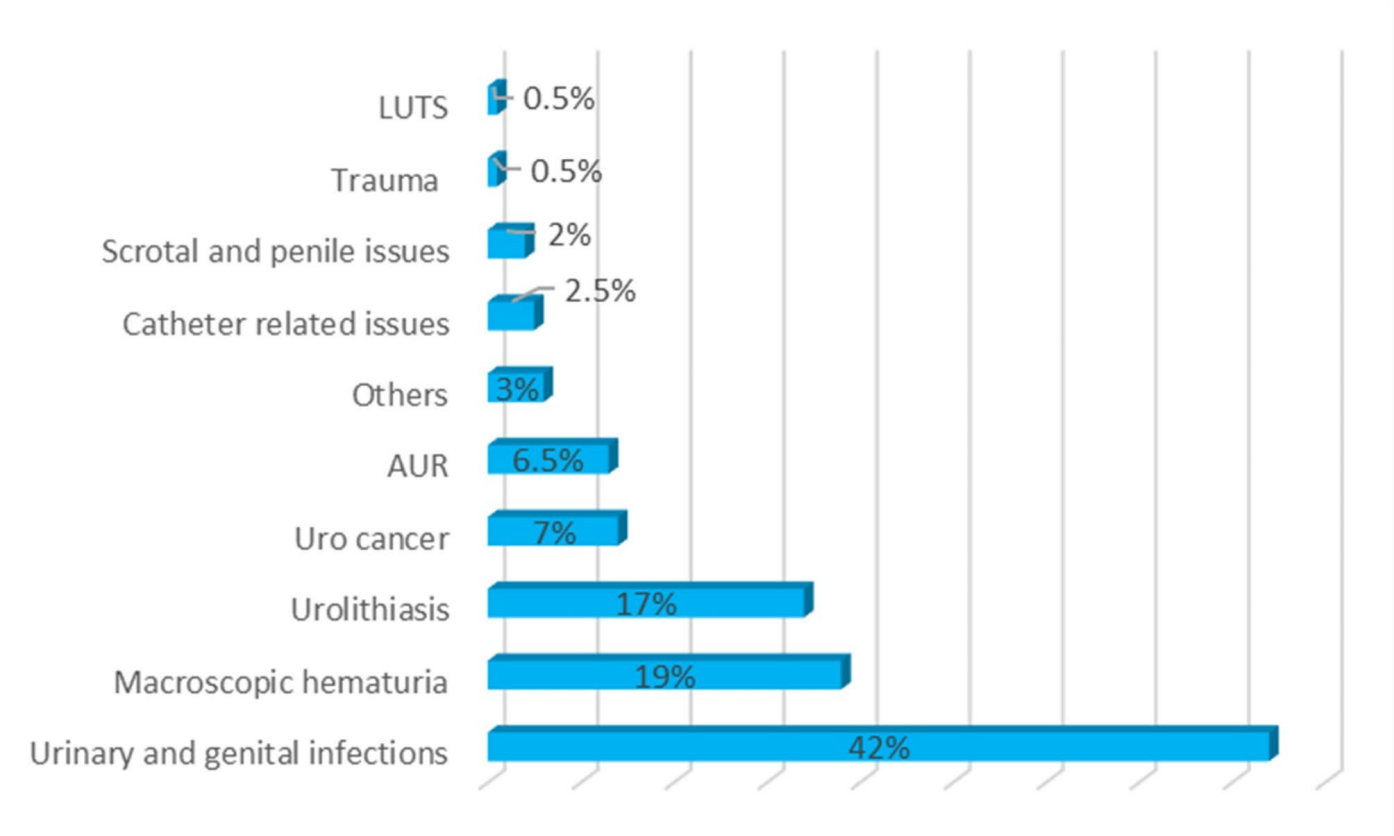
Reasons for admission of patients presenting to the Emergency Department with urological complaints at the NU Hospital Group in Sweden in 2019

Out of the total patient population, 675 patients (28%) required admission to a ward. The most common reason for admission was UGIs (*n* = 285, 42%), followed by macroscopic hematuria (*n* = 114, 19%). The majority of patients were admitted to the urology ward. The median hospital length of stay was 4 days (IQR 3–6). The admission rate varied depending on the mode of referral, with 583 patients (29%) of self-referred patients being admitted, compared to 78 patients (21%) of patients referred by primary care. (Tables 1 and 3; Fig. 3).

**Table 3:** Characteristics of patients with urological catheters (*n* = 491). Figures represent number of patients (% of the row) if not otherwise indicated. (IQR: interquartile range)

Variable name		All
Gender	Male	416 (85)
Age	Years (IQR)	77 (70–86)
Chief complaint	Catheter related issues	137 (28)
	Macroscopic hematuria	118 (24)
	Infectious symptoms	99 (20)
Catheter type*	Indwelling catheter	251 (10)
	Pyelostomy	127 (5)
	Suprapubic catheter	82 (3)
	Double pigtail catheter	40 (2)
Postoperative complication	yes	102 (21)
Admission	All patients	212 (43)
Admission length	Days (IQR)	5 (4–8)
Patients undergone acute procedure	All patients	45 (9)
Radiology	All	185 (38)
Acute treatment	With antibiotics	233 (48)
Number of contacts	1–4	218 (44)
	> 4	65 (13)

* Patients presenting to the emergency department who already have any type of urological catheter in place

Self-referred patients were more likely to have urolithiasis (26%) compared to those who visited GPs first (15%), while those who visited a GP first were more likely to have UGIs (46%) compared to those who went directly to the ED (36%).

### Radiology and treatment

Among the entire cohort, 1 176 patients (48%) underwent radiological investigation either directly at the first ED visit or within 4 weeks. At the initial ED visit, acute radiological imaging was performed in 712 patients (29%). Among them, 464 patients (65%) underwent a CT scan. Among the positive findings, urolithiasis was the most common (*n* = 164, 23%), followed by epididymo-orchitis (*n* = 46, 6.5%) diagnosed mostly with ultrasound. Notably, 466 examinations (65% of radiological examinations) showed no uro-radiological pathological findings.

A total of 215 patients (9%) underwent surgical interventions. Among them, the most frequently performed procedure was endoluminal stone surgery, conducted in 68 patients (32%), followed by catheter adjustments in 47 patients (22%), including adjustments to the pyelostomy catheter, management of indwelling catheter clotting, or the placement of a double pigtail catheter. A total of 999 patients (41%) received antibiotic treatment.

### Patients with urological catheters

Within the cohort, 491 patients (20%) presenting with urological complaints had some form of urological catheter in place, with 14% of them having more than one catheter. The median age for these patients was noticeably higher than the overall cohort 77 years (IQR 70–86), and the majority (*n* = 416, 85%) were male. The primary complaints were blockage, dislodgement, or macroscopic hematuria. Among these patients, 212 (43%) required hospitalization, with a median length of stay of 5 days (IQR 4–8), which was longer compared to 3 days (IQR 2–6) for those without a catheter. Almost half of the patients received antibiotics, and more than half had at least one contact with the urological unit within a year of their ED visit. (Table 3)

## Discussion

In this study, a majority of patients presenting to the ED with urological issues were male. Most patients were triaged to the SUT section, with the majority self-referred. One reason for some patients to not be referred to the SUT section is that urinary infections in females usually is seen as an infectious disease or internal medicine problem in Sweden. Urological symptoms such as loin pain, fever, LUTS and macroscopic hematuria were common, with UGIs being the most frequent diagnoses. A significant portion of patients required admission, particularly for UGIs and macroscopic hematuria, with a median hospital stay of 4 days. Acute radiological imaging was performed in a substantial number of patients, with CT being the most common procedure, though many exams revealed no urological pathological findings.

In contrast to other studies [6, 17] demonstrating seasonal variations in incidence of urological emergencies, our findings showed no evidence of such seasonal variation. This discrepancy may be attributed to the fact that summer in other countries are significantly hotter than in Sweden, making individuals more susceptible to dehydration during the summer months and, consequently, more prone to renal colic. Furthermore, the diagnostic agreement variability, assessed using Cohen’s kappa (κ = 0.741), indicated an elevated level of consistency between the preliminary diagnoses made by ED staff and the final diagnoses established after the evaluation of all radiological and laboratory findings.

Among the 106 patients with four or more contacts with a urological unit during the study period, recurrent presentations were predominantly due to UGIs, macroscopic hematuria, and urolithiasis. These findings align with studies showing that patients with chronic urologic conditions often experience repeated acute episodes that necessitate imaging [18], noted that recurrent stone formers benefit from CT imaging to monitor stone burden and assess for complications such as obstruction or infection, emphasizing the role of imaging in long-term management.

Interestingly, the admission rate was higher among patients who sought care independently compared to those referred by a GP in primary care. This suggests that self-referred patients may present with more severe conditions, as they often contact emergency services directly due to serious symptoms, leading to a higher proportion of severe cases in this group. However, the overall admission rate remains relatively low, with only one-fifth of patients being admitted, compared to the national average, where approximately one in three emergency visits results in hospital admission [19]. The relatively low admission rate, despite the higher number of GP referrals, may indicate that primary care providers might not always fully assess the severity of conditions before referring patients to the ED [20]. This highlights areas where the primary care system could benefit from increased capacity to manage urological emergencies [21].

To address this, targeted training programs that enhance primary care providers’ ability to diagnose, and manage such conditions, including when to appropriately refer patients to specialized care, would be beneficial. Additionally, equipping primary care facilities with essential diagnostic tools and allocating more resources to improve access to primary care physicians would allow patients to receive timely assessments, reducing unnecessary ED visits. Furthermore, improving access to specialist consultations through telemedicine or streamlined referral systems could ensure timely expert advice and reduce unnecessary ED referrals. Additionally, establishing a dedicated catheter nurse-led clinic for patients would be highly beneficial in providing specialized care, improving patient outcomes, and optimizing resource utilization. Overall, only a small proportion of ED visits lead to hospital admission. This suggests that many patients may be seeking emergency care for conditions that could have been effectively managed in a well-equipped primary care setting or through self-care.

Self-referred patients were more likely to have urolithiasis, whereas those who visited a GP before their ED visit more commonly presented with UGIs. This pattern may be influenced by differences in the perceived severity of symptoms and the urgency felt by the patients. Symptoms of urolithiasis, such as severe pain, might prompt individuals to bypass primary care and seek immediate help at the ED. In contrast, UGIs symptoms might initially seem less severe, leading patients to seek care at primary healthcare centers first, where the infection is identified and potentially escalated to the ED if necessary. Additionally, primary care providers may triage and refer UTI cases to the ED more frequently when complications or advanced care are required.

Our study reveals that almost half of all patients underwent acute imaging during their ED visit or within 4 weeks. The high proportion of normal imaging results observed in this study highlights the need for more judicious use of imaging. Over-reliance on imaging may lead to unnecessary radiation exposure [22], increased healthcare costs, and delays in clinical decision-making. Although this high rate of negative results might suggest potential overuse, 23% of cases had urolithiasis. Clinical decision tools, such as the STONE score, or other diagnostic algorithms have been validated to predict the likelihood of urolithiasis and can aid in selecting patients who would benefit most from imaging [23].

To mitigate these risks, it is crucial to establish evidence-based guidelines for when CT imaging is truly necessary [24]. While CT provides superior diagnostic accuracy, US offers a radiation-free alternative but is operator-dependent. Comparative studies, such as those by *Smith-Bindman et al.* [25] suggest that tailored approaches integrating clinical judgment and resource availability can optimize imaging use. Encouraging the use of alternative diagnostic methods, such as US where appropriate, and providing ongoing training for healthcare professionals in clinical decision-making may therefore improve the judicious use of imaging. Emerging technologies and AI-driven imaging algorithms may improve diagnostic accuracy further [26].

Patients with urological catheters constitute a vulnerable subgroup. This population is predominantly elderly, making them more prone to complications. The use of catheters increases the risk of infections and other complications, with nearly half of these patients requiring hospitalization. In accordance with our results, urinary catheterization is highly prevalent among patients admitted to urology departments, with up to 75% receiving a catheter during their hospital stay and around 20% already having one in place before admission [27].

The frequent need for hospital admission highlights the complexity of catheterized patients’ conditions. Additionally, nearly half of these patients received antibiotic treatment, indicating a high burden of infection or infection-related concerns in this population [28]. This underscores the need for better preventive measures, such as improved catheter care protocols, timely assessments, and judicious use of antibiotics to prevent resistance.

Our study has several strengths, being the first Scandinavian investigation regarding the epidemiology of urological emergencies. It provides valuable insights into the urological acute care landscape and related factors within the population, offering a snapshot of data that can inform public health planning and resource allocation. Moreover, with a study population of 2 433 patients, it provides a solid foundation for making further decisions regarding the development of acute care services. However, there are limitations to consider. Given the cross-sectional nature of the study, it cannot establish causal relationships, as both exposure and outcomes are measured at the same time. Additionally, the study may be influenced by biases, including selection bias that the participants included in this study may not be representative of the broader population, potentially skewing the results and limiting the generalizability of the findings.

## Conclusion

Most patients self-referred to the ED, and many required admissions, particularly for UGIs. Acute imaging, mostly CT scan, was performed in a considerable number of cases, although many revealed no pathological findings. Interestingly, self-referred patients had a higher admission rate compared to those referred by a GP, suggesting potential gaps in the primary healthcare system. Enhancing the management of urological emergencies in primary care and establishing clearer guidelines for acute radiological imaging could play a significant role in improving patient outcomes.

## Data Availability

No datasets were generated or analysed during the current study.

## Abbreviations

AUR: Acute urinary retention
CT: Computed tomography
ED: Emergency department
GP: General practitioner
ICD10: The international classification of diseases
LUTS: Lower urinary tract symptoms
SUT: Section for surgical, urological, and trauma patients
UGI: Urinary and genital infection
US: Ultrasound
